# Therapeutic efficiency of the APAF‐1 antagonist LPT99 in a rat model of cisplatin‐induced hearing loss

**DOI:** 10.1002/ctm2.363

**Published:** 2021-04-05

**Authors:** Silvia Murillo‐Cuesta, Adelaida M Celaya, Blanca Cervantes, Jose M Bermúdez‐Muñoz, Lourdes Rodríguez‐de la Rosa, Julio Contreras, Isabel Sánchez‐Pérez, Isabel Varela‐Nieto

**Affiliations:** ^1^ Biomedical Research Networking Center on Rare Diseases (CIBERER) Institute of Health Carlos III Madrid Spain; ^2^ Institute for Biomedical Research “Alberto Sols” Spanish National Research Council‐Autonomous University of Madrid Spain; ^3^ Hospital La Paz Institute for Health Research Madrid Spain; ^4^ Anatomy and Embriology Department, Faculty of Veterinary Universidad Complutense de Madrid Madrid Spain; ^5^ Biochemistry Department Faculty of Medicine Autonomous University of Madrid Madrid Spain

Dear Editor,

Cis‐diammine‐dichloroplatinum[II] (cisplatin) is a potent and widely used chemotherapeutic agent with significant efficacy against several forms of cancer in adults and children. Unfortunately, cisplatin has multiple adverse effects and causes irreversible hearing loss (HL) in a large proportion of patients, which severely impacts their quality of life.^1^ Here, we confirm that treatment with LPT99, a small molecule inhibitor of the apoptosome, preserves hearing levels in a rat model of cisplatin‐induced HL and prevents apoptosis in cisplatin‐exposed HEI‐OC1 cells. These data are encouraging and support the potential of LPT99 for the prevention of the secondary effects of cisplatin for HL.

Cisplatin primarily induces damage to outer (OHC) and inner hair cells and to spiral ganglion neurons, and long‐term retention of cisplatin in the cochlea contributes to its irreversible ototoxic effects.^2^ Once inside the cell, cisplatin forms a highly reactive complex that induces DNA damage, activates death receptor pathways, and causes mitochondrial dysfunction, triggering reactive oxygen species generation, oxidative stress, and cytochrome c release. Cytochrome c binds with apoptotic protease activating factor 1 (APAF‐1) forming the apoptosome, which recruits and activates caspase‐9 and ‐3 to execute apoptosis^3^ (Figure 1A). Antioxidants and antiapoptotic drugs have been tested as otoprotectants in experimental models and clinical trials^4^ (Figure 1A), but their lack of specificity and efficacy are major limitations and none are currently approved for the treatment of cisplatin‐induced HL. Accordingly, more effective agents are urgently needed, preferably formulated for local (intratympanic) administration to avoid interfering with the antineoplastic activity of cisplatin.

**FIGURE 1:**
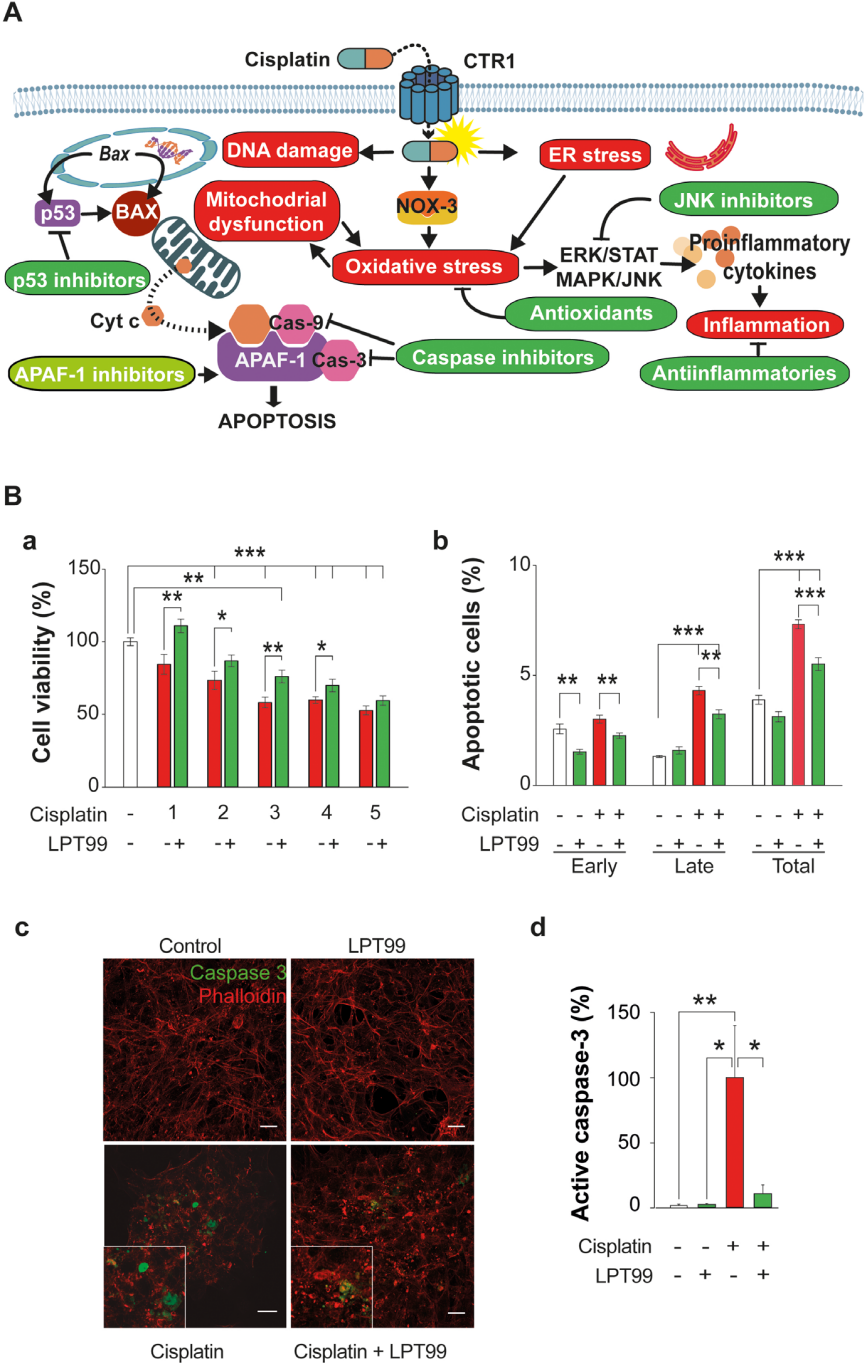
(A) General mechanisms of cisplatin‐induced cytotoxicity. Cisplatin enters into the cell by passive diffusion or by active uptake through specialized copper transporters. Once in the cytosol, cisplatin forms a highly reactive complex that activates several pathological molecular processes (red ovals) including reactive oxygen species (ROS) production, DNA damage, BAX translocation to the mitochondria, and cytochrome c (Cyt c) release. Apoptosome formation is triggered by Cyt c binding to the apoptotic protease activating factor 1 (APAF‐1) in the cytosol along with caspase‐9 and ‐3 (Cas‐9 and Cas‐3) recruitment. Apoptosis inhibitors and other blocking molecules (green ovals) have been tested as otoprotective candidates. Abbreviations: CRT1, copper receptor transporter 1; NOX‐3, NADPH oxidase 3; ER, endoplasmic reticulum. (B) LPT99 suppresses cisplatin‐induced apoptosis in HEI‐OC1 cells. (a) Cell viability in HEI‐OC1 cells untreated (control, white bar), challenged with cisplatin (1–5 μg/ml, red bars) or co‐treated with LPT99 (1 μM, green bars), and cultured for 24 h. Data are expressed as % relative to the control (100% viability) and shown as mean ± SEM of two independent experiments, performed in quadruplicate. (b) Apoptosis in HEI‐OC1 cells untreated (control, white bars), challenged with cisplatin (3 μg/ml, red bars) or co‐treated (1 μM, green bars) with LPT99 and incubated for 24 h. Data are expressed as the percentage of apoptotic cells (early, late or total) in the plate and expressed as mean ± SEM of two independent experiments, performed in triplicate. (c) Microphotographs of HEI‐OC1 cells untreated (control) or treated with LPT99 (1 μM), cisplatin (4 μg/ml) or both for 24 h, and immunostained for activated caspase‐3 (green) and phalloidin (red). Scale bar 20 μm. Images are representative of triplicate samples from two independent experiments and quantified in (d). (d) Active caspase‐3 staining of HEI‐OC1 cells untreated (control, white bar), challenged with cisplatin (red) and co‐treated (green) or not with LPT99, expressed as the percentage of relative to the cisplatin group. Statistical differences were assessed by one‐way ANOVA and Student's *t*‐test, or the Kruskal–Wallis and Mann–Whitney *U* test, for normal or nonnormal data, respectively. Differences were considered significant for *p < 0.05, **p < 0.01, ***p < 0.01

As a central component of the apoptosome and a regulator of apoptosis, APAF‐1 is a key pharmacological target that has been validated in experimental models.^5^ Orzáez et al.^6^ described a series of 2,5‐piperazinedione derivatives (Patent US9040701B2) with APAF‐1 inhibitory activity through binding to the APAF‐1 caspase recruitment and nucleotide‐binding oligomerization domains, which impair the conformational change required for its oligomerization and, consequently, inhibit the activation of procaspase‐9 and the induction of apoptosis. These molecules attenuated cisplatin‐induced apoptosis in HEI‐OC1 cells and the loss of neuromasts in zebrafish, demonstrating that APAF‐1 inhibition is effective against cisplatin‐associated ototoxicity.^6^ We investigated the efficacy of LPT99 (Patent US10561736B1), a second‐generation derivative with similar APAF‐1 inhibitory activity but better pharmacological properties, in preventing cisplatin‐induced ototoxicity using in vitro and in vivo models.

The addition of 1 μM LPT99 to cisplatin‐treated HEI‐OC1 cell cultures^7^ significantly improved cell survival (*p* < 0.05) across the cisplatin concentration ranging from 1 to 4 μg/ml (Figure 1B, a). Flow cytometry analysis of annexin V/propidium iodide staining indicated that LPT99 alone did not trigger apoptosis, but significantly reduced the percentage of total apoptotic cells (early and late apoptosis) in co‐treated cultures as compared with cisplatin alone (*p* < 0.001) (Figure 1B, b). Activated caspase‐3 levels were also lower in cells co‐treated with cisplatin and LPT99 (Figure 1B, c and d).

LPT99 was formulated in cyclodextrin and Poloxamer 407 hydrogels to achieve sustained delivery, and was administered intratympanically^8^ in rats. Dosing of LPT99/cyclodextrin (50–200 μM) provided high LPT99 cochlear levels (Figure 2A, a) and had no effect on hearing thresholds even 30 days after administration (Figure 2A, b). Similarly, dosing of LPT99/Poloxamer 407 (100–797 μM) reached the cochlea, was detectable up to 14 days after injection, and had no effect on hearing, supporting its tolerability at the tested doses (Figure 2B). LPT99 was undetectable in plasma at all‐time points and doses studied (quantification limit 5 ng/ml). We evaluated the efficacy of LPT99 by monitoring the auditory brainstem response (ABR) before and 3 days after bilateral intratympanic administration of LPT99 in cyclodextrin (Figure 2A, c) or in Poloxamer 407 (Figures 2C and 3) and intraperitoneal infusion of cisplatin (10 mg/kg). Analysis of the untreated group revealed that 86% of rats exhibited HL following cisplatin administration, whereas normal hearing was observed in 50%, 58%, and 70% of rats in the groups co‐treated with 50, 100, or 478 μM LPT99/Poloxamer 407, respectively (Figure 2C). LPT99 treatment also notably reduced the percentage of animals with moderate and profound HL (Figure 2C). Co‐administration of LPT99 reduced the cisplatin‐induced threshold shifts for clicks (∼50% reduction) and pure tones (∼60%, 45%, and 35% reduction for 8, 16, and 28–40 kHz). Significant dose‐dependent differences were found in threshold shifts between LPT99/Poloxamer 407‐treated and nontreated rats, with the best protection profile observed at a dose between 100 μM and 478 μM LPT99 (Figure 3A and B). Cisplatin‐induced changes in the latency and amplitude of the ABR waves were also attenuated by LPT99 (Figure 3C and D). The LPT99 dosing scheme improved hearing thresholds in a range similar to that reported for other otoprotective drugs, such as a p53 inhibitor,^9^ and improved upon those reported for the intracochlear perfusion of inhibitors of JNK, caspases‐3, 9, and 8 and cathepsin B.^10^

**FIGURE 2:**
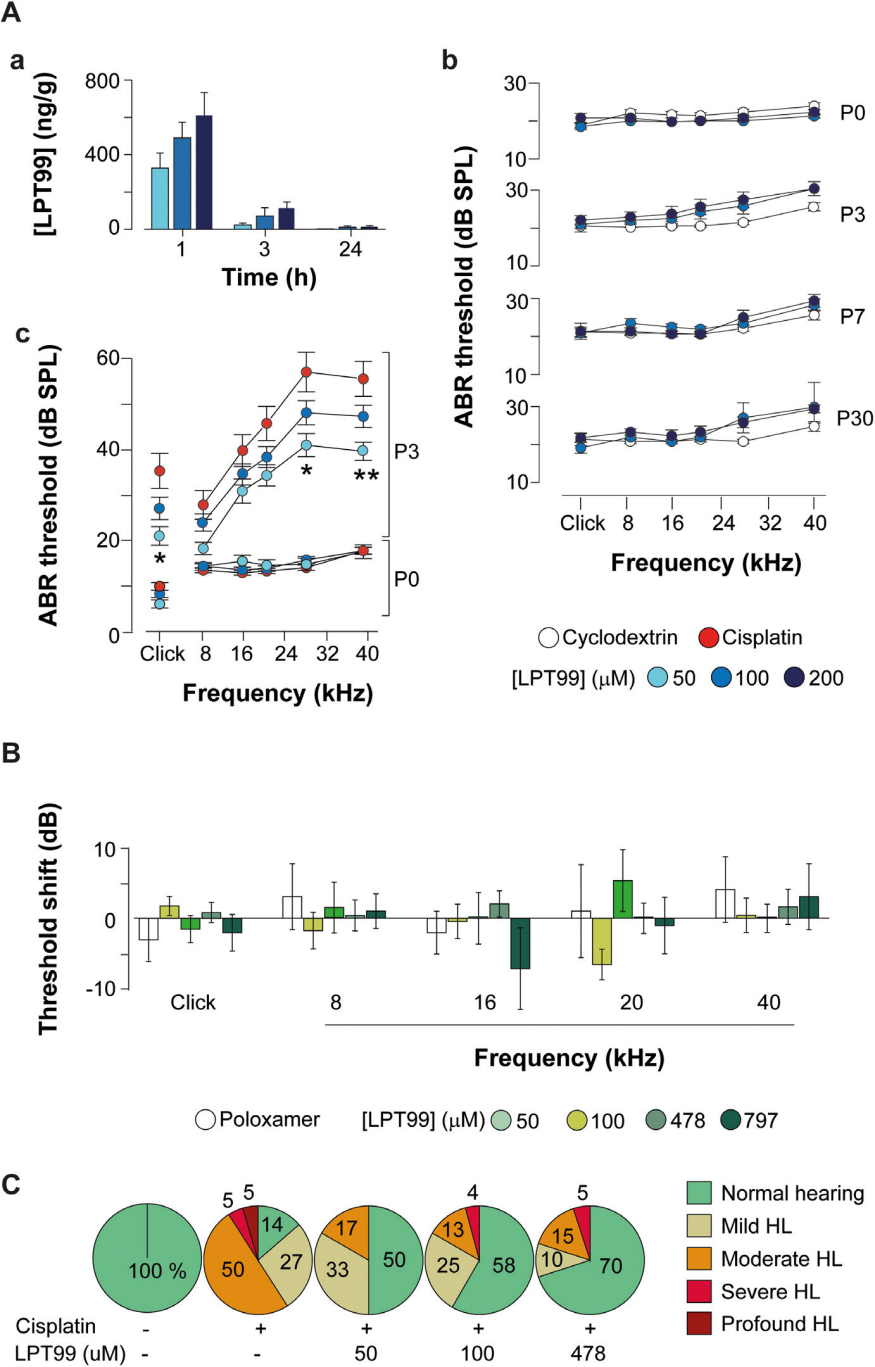
(A) LPT99/cyclodextrin does not modify hearing parameters and prevents cisplatin ototoxicity. (a) LPT99 was evaluated in rat cochleae following intratympanic administration (1, 3, and 24 h postinjection; three animals per time point and product/concentration) of the indicated doses: 50 μM (light blue), 100 μM (blue), and 200 μM (dark blue) in vehicle (5% 2‐hydroxypropyl‐β‐cyclodextrin in saline serum). Results are expressed as mean ± SEM (*n* = 4 cochleae from two animals per time point and concentration). (b) ABR thresholds in response to click and tone bursts stimuli before (baseline, P0) and 3, 7, and 30 days (P3, P7, P30) after bilateral local administration thresholds (left and right ears are shown together) of vehicle (white circles, *n* = 9), 100 μM (blue, *n* = 10) or 200 μM (dark blue, *n* = 10) LPT99. (c) The efficacy of LPT99/cyclodextrin to prevent cisplatin ototoxicity was evaluated by ABR. ABR thresholds (left and right ears are shown together) in response to tone bursts at baseline (P0) and 3 days after (P3) intraperitoneal infusion of cisplatin (10 mg/kg), in rats untreated (red circles, *n* = 17) or treated with LPT99 at 50 (light blue, *n* = 14) or 100 μM (blue, *n* = 24). Note that 50 μM LPT99 attenuated the ABR threshold shifts observed 3 days after cisplatin administration and this was significant for higher frequencies. Results are expressed as mean ± SEM; ANOVA with Bonferroni or Tamhane post hoc tests was used to assess significance, *p < 0.05, **p < 0.01, LPT99 50 μM versus vehicle or cisplatin. (B) Intratympanic LPT99/Poloxamer 407 does not modify hearing parameters. Poloxamer 407 formulation provides sustained drug delivery to the cochlea after intratympanic administration, and allows solubilization of higher concentrations of LPT99 (up to ∼800 μM), making it a more suitable vehicle to treat cisplatin ototoxicity. Safety of the LPT99/Poloxamer 407 formulation was evaluated by ABR recordings before (baseline) and 3 days after intratympanic administration of vehicle (Poloxamer 407, white) (*n* = 9) or increasing doses of LPT99: 100 (*n* = 15), 300 (*n* = 4), 478 (*n* = 16), and 797 (*n* = 4) μM), represented in a green color scale. Results revealed no significant differences in click or tone burst (8–40 kHz) ABR threshold shifts (both ears are shown grouped together) between the experimental conditions tested. Data are expressed as mean ± SEM. Statistical differences were assessed by ANOVA with Bonferroni or Tamhane post hoc test among groups. (C) LPT99/Poloxamer 407 reduces hearing loss severity in cisplatin‐injected rats. Percentage of animals showing normal hearing (averaged ABR thresholds < 46 dB SPL) or hearing loss in each experimental group. Hearing loss was classified as mild (46–55 dB SPL), moderate (56–65 dB SPL), severe (66–75 dB SPL), or profound (>75 dB SPL) hearing loss according to hearing threshold data obtained by ABR. Rats (*n* = 10–11 per group) were intraperitoneally infused with 10 mg/kg cisplatin and co‐treated or not with 50, 100, or 478 μM LPT99

**FIGURE 3:**
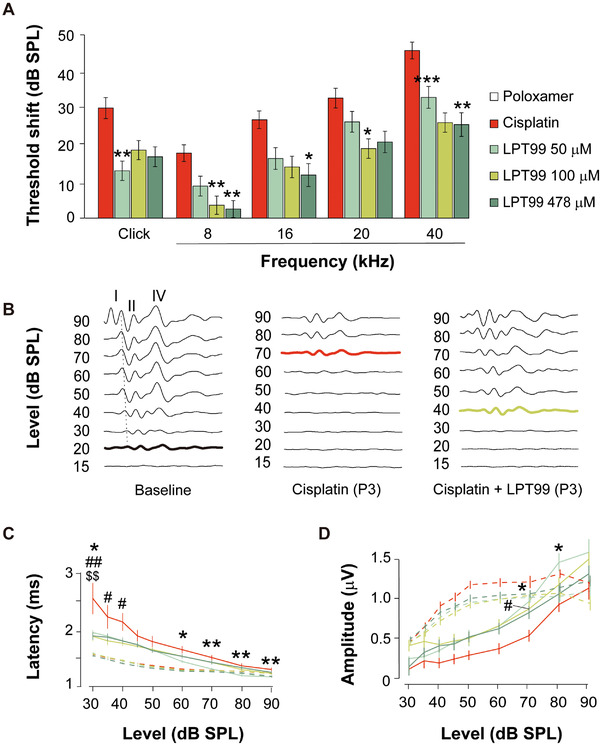
(A) LPT99/Poloxamer 407 decreases cisplatin‐induced threshold shifts. ABR threshold shifts (left and right ears are shown together) in response to click and 8–40 kHz tone bursts stimuli 3 days after systemic cisplatin administration in rats untreated (*n* = 11) (red bars) or treated with LPT99/Poloxamer 407 at 50 (*n* = 6), 100 (*n* = 12), and 478 (*n* = 10) μM. Data are expressed as mean ± SEM. Statistical differences were assessed by ANOVA with Bonferroni or Tamhane post hoc test among groups (*p < 0.05, **p < 0.01, ***p < 0.001 vs. untreated group). (B) Representative ABR recordings in response to a click stimulus before (baseline) and 3 days after cisplatin administration (P3), from an untreated rat with severe hearing loss and an LPT99‐cotreated rat with mild hearing loss. ABR waves are indicated in Latin numbers (I to V) and thresholds are highlighted in red and green bold lines for untreated and treated rats, respectively. (C and D) LPT99/Poloxamer 407 reduces cisplatin‐induced changes in ABR latencies and amplitudes. Input–output functions of ABR wave I in response to click stimulus. Latency (C) and amplitude (D) mean values are plotted against sound intensity (in dB SPL). ABR data from left and right ears are shown together. Experimental groups untreated (red) or treated with LPT99 at 50 (*n* = 6), 100 (*n* = 12), and 478 (*n* = 10) μM are shown before (baseline) and 3 days after cisplatin administration. Data are expressed as mean ± SEM. Statistical differences between untreated group were assessed by ANOVA with Bonferroni or Tamhane post hoc test (* vs. LPT99 at 50 μM; # vs. LPT99 100 μM; $ vs. LPT99 478 μM. One symbol indicates *p* < 0.05, two symbols indicate *p* < 0.01)

The protective effects of LPT99/Poloxamer 407 were further confirmed by assessing cochlear cytoarchitecture (Figure 4). Cisplatin is known to induce substantial OHC loss in the basal and middle turns. Rats co‐treated with LPT99 showed better preservation of the organ of Corti and spiral ganglion (Figure 4A–F, J, and L). The protective effect of LPT99 against cisplatin‐induced OHC loss was further confirmed by the preserved E‐cadherin and F‐actin expression (Figure 4G, K, M, and Q) and the higher expression levels of prestin, a motor OHC protein (Figure 4H). LPT99‐co‐treated cochleae also showed less dilatation of strial microvasculature, particularly in the middle turn and exhibited higher levels of Kir4.1, a marker of intermediate cells (Figure 4I and O). Because the stria vascularis is the entry point of cisplatin into the cochlea and a long‐term accumulation site, it has emerged as an important target to prevent cisplatin ototoxicity.^2^ Further studies are needed to demonstrate a direct effect of LPT99 on strial cells.

**FIGURE 4:**
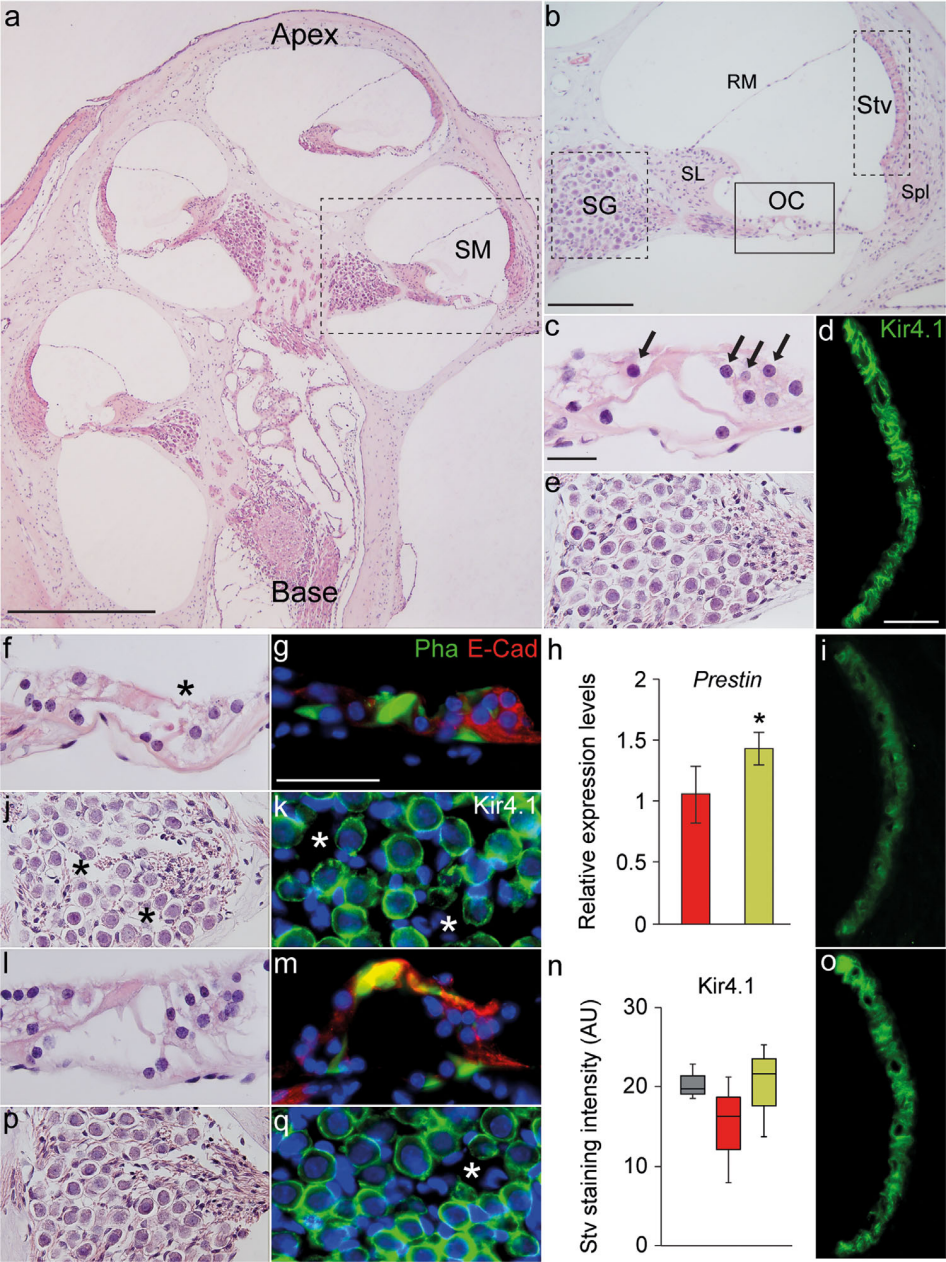
LPT99 protects the cochlea against cisplatin‐induced structural damage. Representative images of cochlear cytoarchitecture from control (A–E), cisplatin‐injected (F–G and I–K) and LPT99/Poloxamer 407 (100 μM)‐treated (L, M, and O–Q) rats. Scala media (dashed line box in A). Spiral ganglion (dashed line box in B), magnifications shown in E, J, and P. Organ of Corti (continuous line box in B), magnifications shown in C, F, and L. Stria vascularis (dashed line rectangle in B), magnifications shown in D, I, and O. Arrows in C indicate hair cells. Asterisks in K and Q indicate absence of staining. Scale bars: 500 μm in A, 200 μm in B, and 50 μm in C, D, and G. (H) qPCR of *Prestin* (*Slc26a5*) expression was calculated as 2^–ΔΔCt^ relative to *Hprt1* and normalized to data from cisplatin‐treated rats (LPT99/Poloxamer 407 100 μM, green; cisplatin, red). Values are presented as mean ± SEM. Statistical significance between groups (*n* = 3/group) was analyzed by Student's *t*‐test (*p < 0.05; **p < 0.01; ***p < 0.001). (N) Boxplot representation of Kir4.1 staining intensity in stria vascularis of LPT99/Poloxamer 407 (100 μM) treated (green), cisplatin‐injected (red) rats, and control rats (gray). Abbreviations: OC, organ of corti; RM, Reissner membrane; SG, spiral ganglion; SL, spiral Limbus; SM, scala media; Spl, spiral ligament; Stv, stria vascularis

In conclusion, LPT99 exerts dose‐dependent protective effects on auditory function and cochlear cytoarchitecture in cisplatin‐treated rats. LPT99 also protects HEI‐OC1 cells against cisplatin‐induced apoptosis, although further mechanistic studies will be needed to fully establish the participation of APAF‐1. Overall, our findings highlight the role of APAF‐1 as a pharmacological target for new drugs and point to LPT99 as a potential prophylactic local treatment for cisplatin‐induced HL.

## CONSENT FOR PUBLICATION

All authors read and approved the final manuscript.

## DATA AVAILABILITY STATEMENT

The data that support the findings of this study are available on request from the corresponding author. The data are not publicly available due to privacy or ethical restrictions.

## CONFLICT OF INTEREST

SMC has received research support from Spiral Therapeutics and IVN has received compensation for consulting. The remaining authors declare that they have no significant competing financial, professional, or personal interests that might have influenced the performance or presentation of the work described in this manuscript.

## FUNDING INFORMATION

Centro de Investigación Biomédica en Red de Enfermedades Raras (CIBERER‐SPIRALTH ER17PE12); Seventh Framework Programme, People: Marie‐Curie Actions, Industry‐Academia Partnerships (FP7‐PEOPLE‐2013‐IAPP‐TARGEAR); European Regional Development Fund, Spanish Ministerio de Economía y Competitividad (SAF2017‐86107‐R)

## AUTHOR CONTRIBUTIONS

Participated in research design: Silvia Murillo‐Cuesta and Isabel Varela‐Nieto; conducted experiments: Silvia Murillo‐Cuesta, Blanca Cervantes, Lourdes Rodríguez de la Rosa, Jose M. Bermúdez‐Muñoz, Julio Contreras, and Adelaida M. Celaya; performed data analysis: Silvia Murillo‐Cuesta, Blanca Cervantes, Lourdes Rodríguez de la Rosa, Adelaida M. Celaya, Isabel Sánchez‐Pérez, and Isabel Varela‐Nieto; wrote or contributed to the writing of the manuscript: Silvia Murillo‐Cuesta, Isabel Sánchez‐Pérez, and Isabel Varela‐Nieto.
